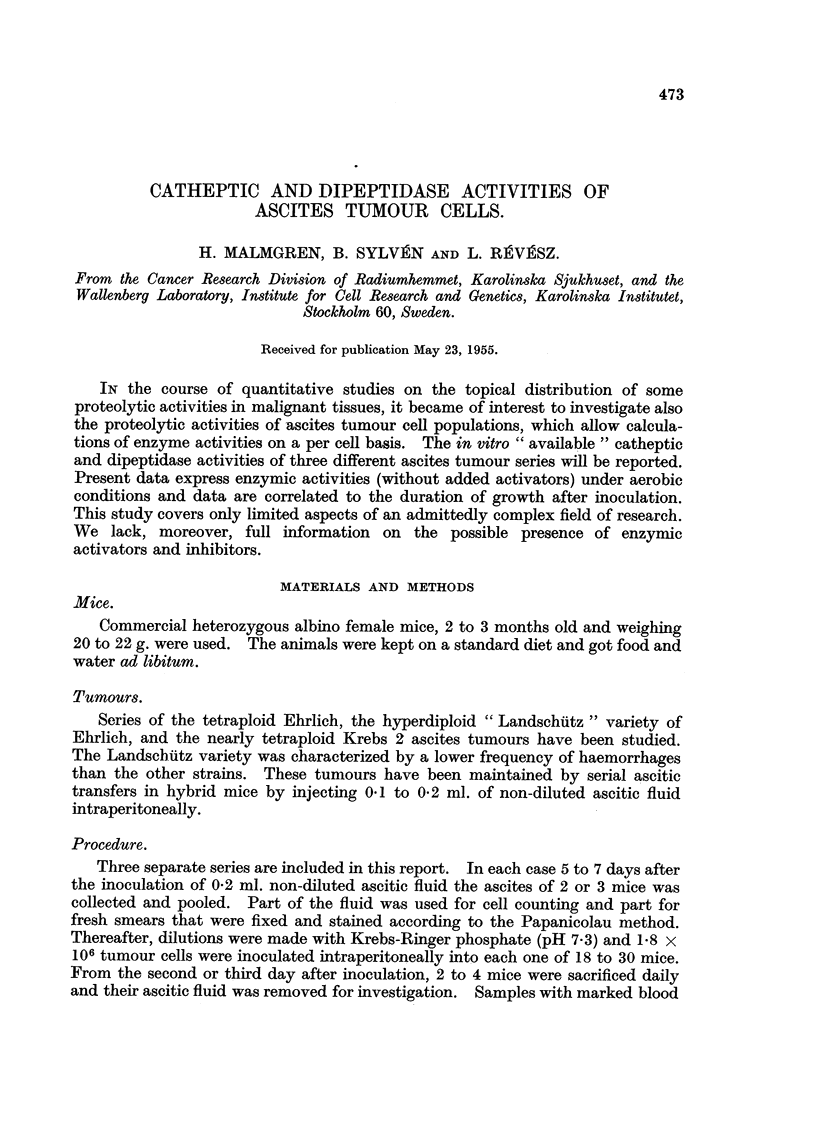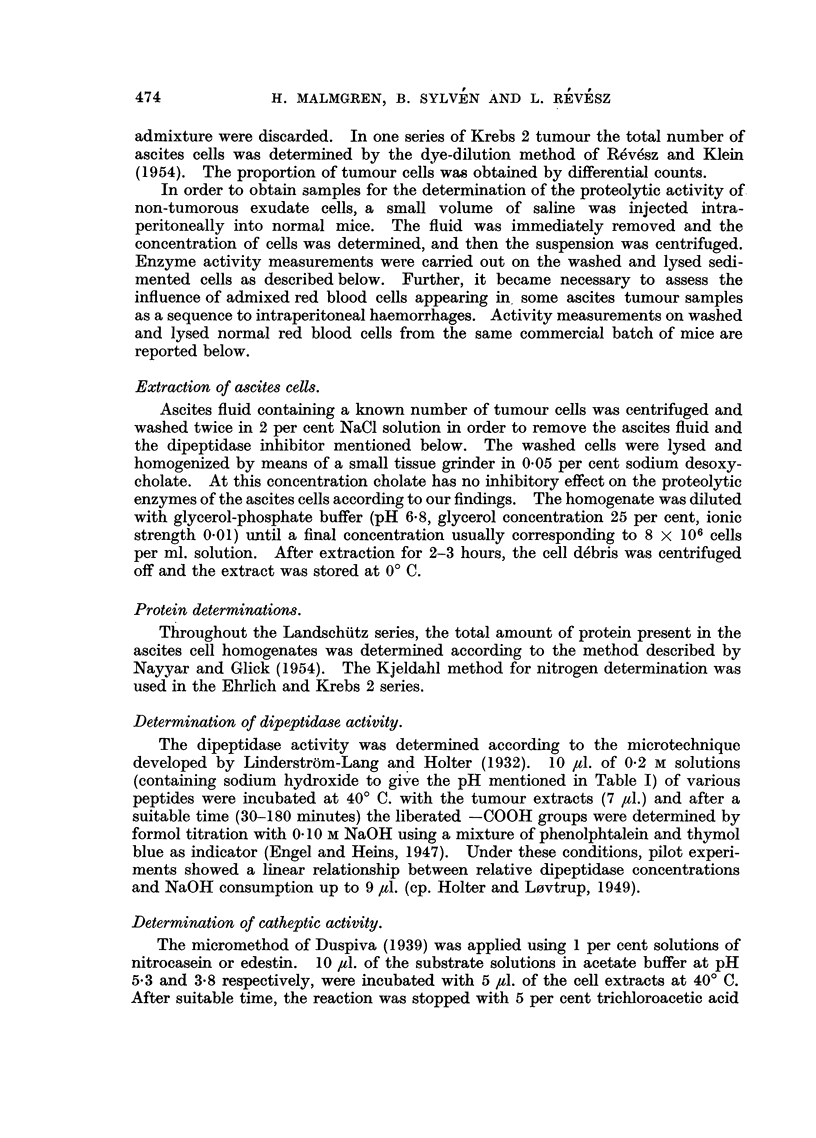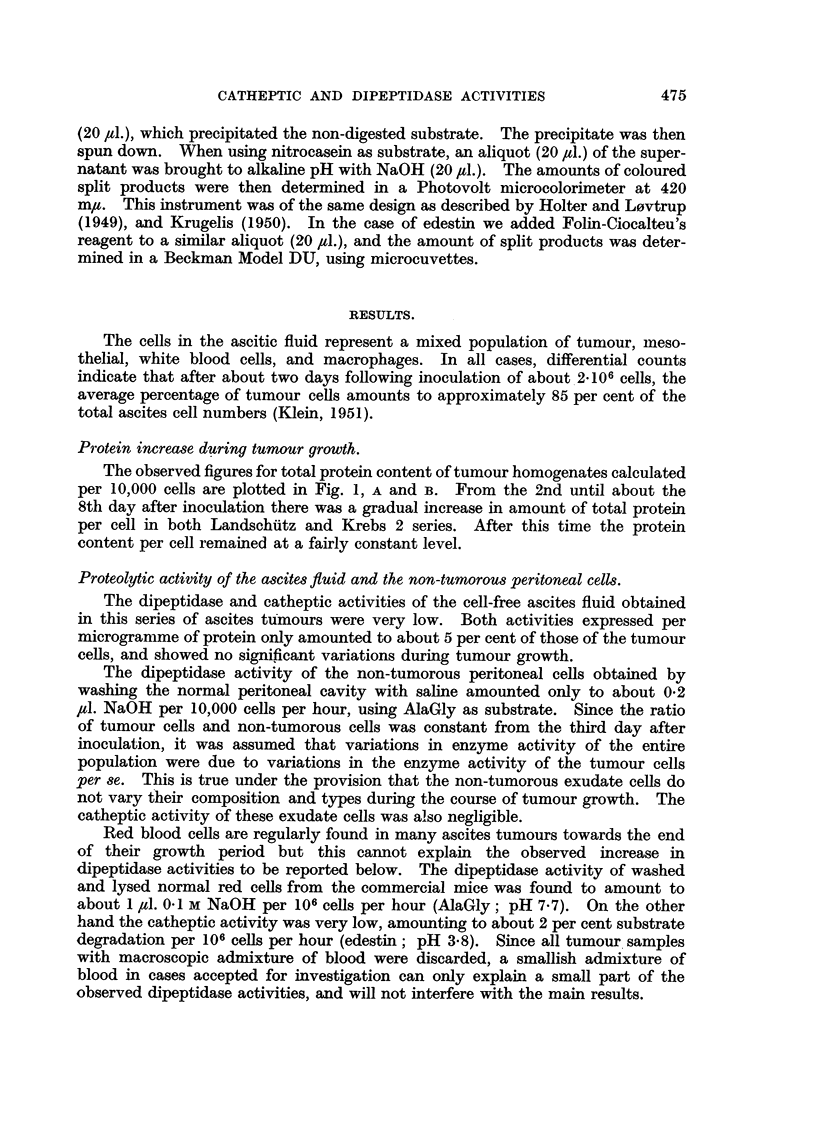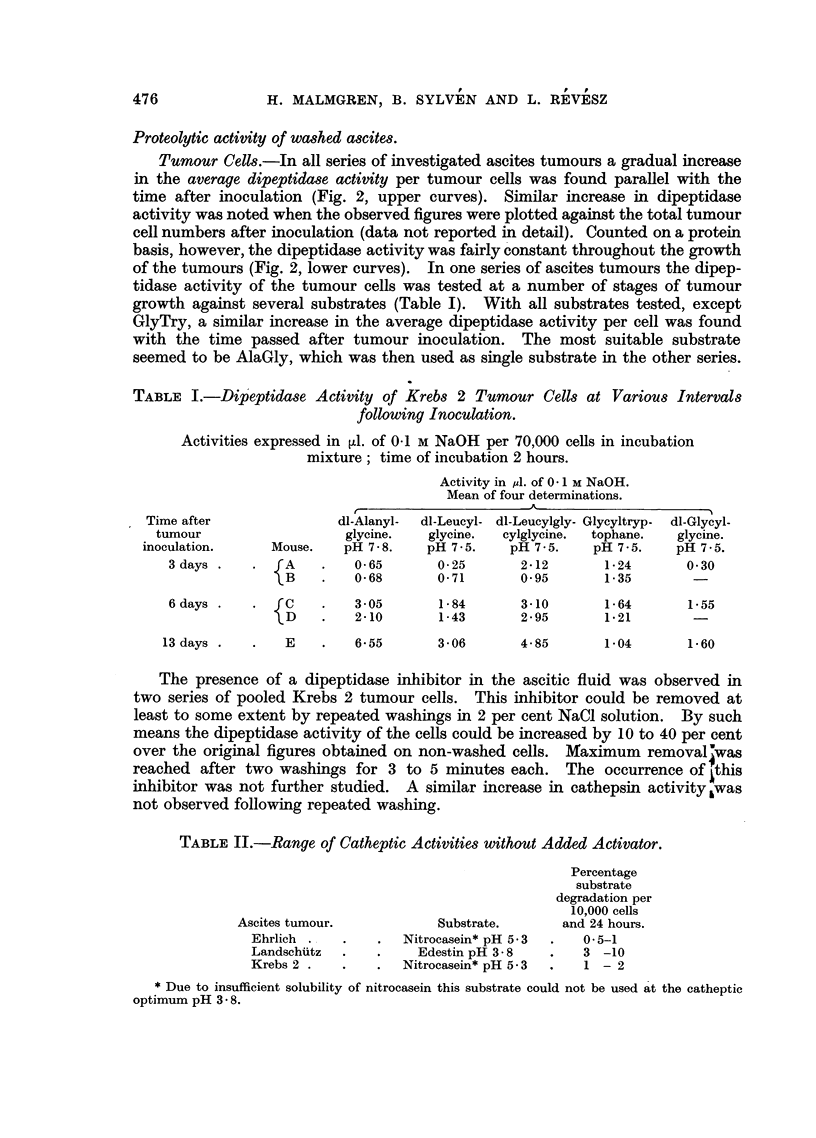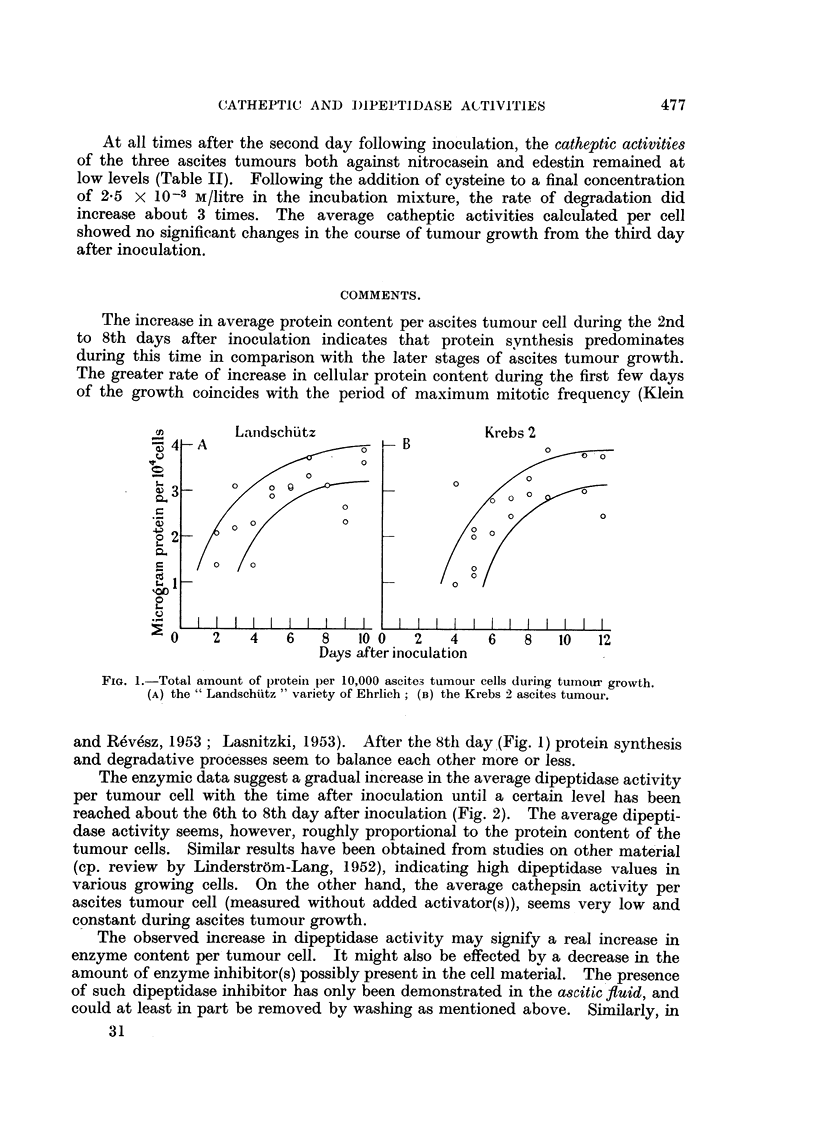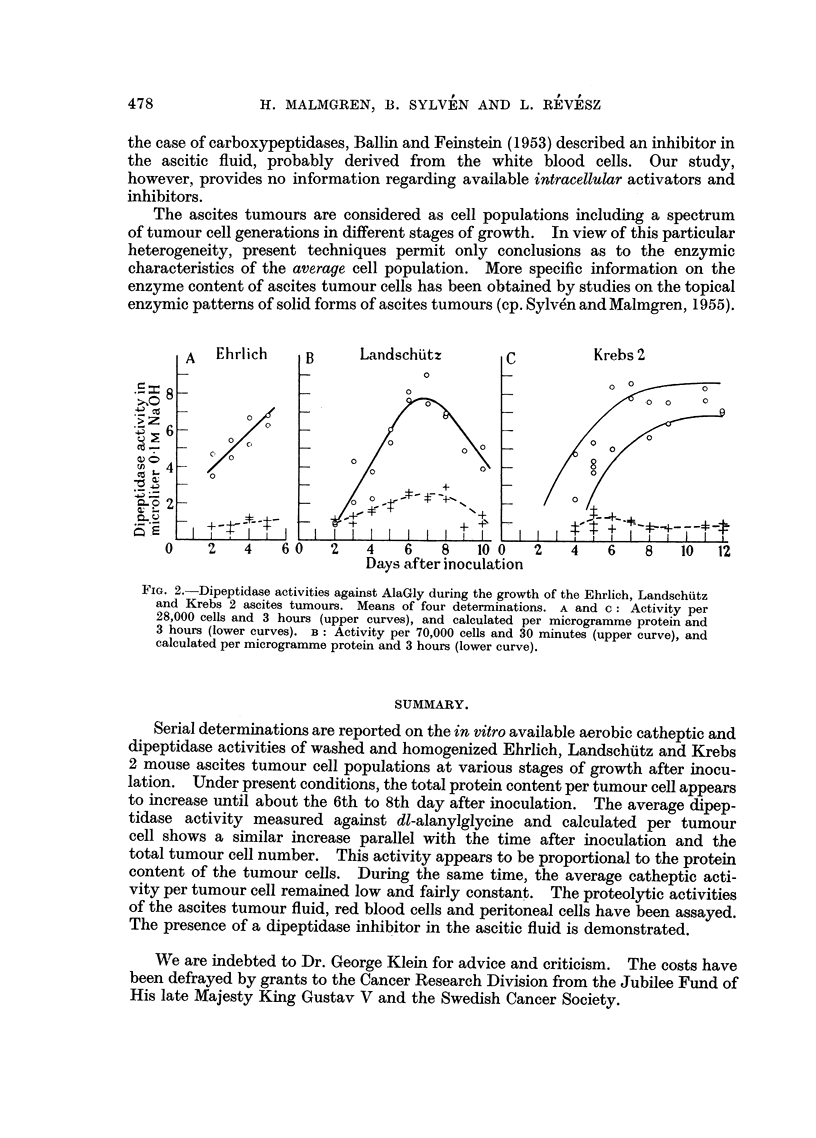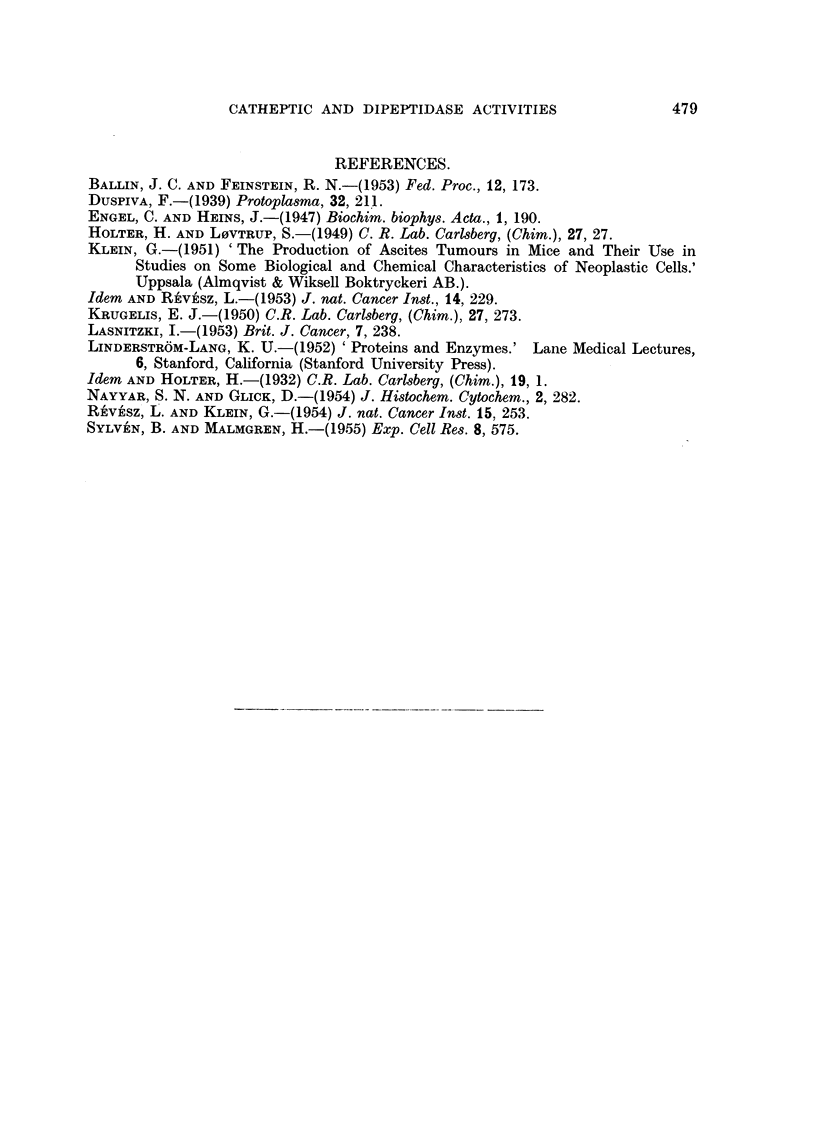# Catheptic and Dipeptidase Activities of Ascites Tumour Cells

**DOI:** 10.1038/bjc.1955.49

**Published:** 1955-09

**Authors:** H. Malmgren, B. Sylvén, L. Révész


### CATHEPTIC AND DIPEPTIDASE ACTIVITIES OF ASCITES TUMOUR CELLS.

H. MALMGREN, B. SYLVÉN AND L. RÉVÉSZ.

*From the Cancer Research Division of Radiumhemmet, Karolinska Sjukhuset, and the Wallenberg Laboratory, Institute for Cell Research and Genetics, Karolinska Institutet, Stockholm* 60, *Sweden.*

Received for publication May 23, 1955.

In the course of quantitative studies on the topical distribution of some proteolytic activities in malignant tissues, it became of interest to investigate also the proteolytic activities of ascites tumour cell populations, which allow calculations of enzyme activities on a per cell basis. The *in vitro* " available " catheptic and dipeptidase activities of three different ascites tumour series will be reported. Present data express enzymic activities (without added activators) under aerobic conditions and data are correlated to the duration of growth after inoculation. This study covers only limited aspects of an admittedly complex field of research. We lack, moreover, full information on the possible presence of enzymic activators and inhibitors.

### MATERIALS AND METHODS

### *Mice.*

Commercial heterozygous albino female mice, 2 to 3 months old and weighing 20 to 22 g. were used. The animals were kept on a standard diet and got food and water *ad libitum.*

### *Tumours.*

Series of the tetraploid Ehrlich, the hyperdiploid " Landschütz " variety of Ehrlich, and the nearly tetraploid Krebs 2 ascites tumours have been studied. The Landschütz variety was characterized by a lower frequency of haemorrhages than the other strains. These tumours have been maintained by serial ascitic transfers in hybrid mice by injecting 0·1 to 0·2 ml. of non-diluted ascitic fluid intraperitoneally.

### *Procedure.*

Three separate series are included in this report. In each case 5 to 7 days after the inoculation of 0·2 ml. non-diluted ascitic fluid the ascites of 2 or 3 mice was collected and pooled. Part of the fluid was used for cell counting and part for fresh smears that were fixed and stained according to the Papanicolau method. Thereafter, dilutions were made with Krebs-Ringer phosphate (pH 7·3) and $1{\cdot}8 \times 10^6$ tumour cells were inoculated intraperitoneally into each one of 18 to 30 mice. From the second or third day after inoculation, 2 to 4 mice were sacrificed daily and their ascitic fluid was removed for investigation. Samples with marked blood admixture were discarded. In one series of Krebs 2 tumour the total number of ascites cells was determined by the dye-dilution method of Révész and Klein (1954). The proportion of tumour cells was obtained by differential counts.

In order to obtain samples for the determination of the proteolytic activity of non-tumorous exudate cells, a small volume of saline was injected intraperitoneally into normal mice. The fluid was immediately removed and the concentration of cells was determined, and then the suspension was centrifuged. Enzyme activity measurements were carried out on the washed and lysed sedimented cells as described below. Further, it became necessary to assess the influence of admixed red blood cells appearing in some ascites tumour samples as a sequence to intraperitoneal haemorrhages. Activity measurements on washed and lysed normal red blood cells from the same commercial batch of mice are reported below.

*Extraction of ascites cells.*

Ascites fluid containing a known number of tumour cells was centrifuged and washed twice in 2 per cent NaCl solution in order to remove the ascites fluid and the dipeptidase inhibitor mentioned below. The washed cells were lysed and homogenized by means of a small tissue grinder in 0·05 per cent sodium desoxycholate. At this concentration cholate has no inhibitory effect on the proteolytic enzymes of the ascites cells according to our findings. The homogenate was diluted with glycerol-phosphate buffer (pH 6·8, glycerol concentration 25 per cent, ionic strength 0·01) until a final concentration usually corresponding to $8 \times 10^6$ cells per ml. solution. After extraction for 2–3 hours, the cell débris was centrifuged off and the extract was stored at 0° C.

*Protein determinations.*

Throughout the Landschütz series, the total amount of protein present in the ascites cell homogenates was determined according to the method described by Nayyar and Glick (1954). The Kjeldahl method for nitrogen determination was used in the Ehrlich and Krebs 2 series.

*Determination of dipeptidase activity.*

The dipeptidase activity was determined according to the microtechnique developed by Linderström-Lang and Holter (1932). 10 $\mu$l. of 0·2 M solutions (containing sodium hydroxide to give the pH mentioned in Table I) of various peptides were incubated at 40° C. with the tumour extracts (7 $\mu$l.) and after a suitable time (30–180 minutes) the liberated —COOH groups were determined by formol titration with 0·10 M NaOH using a mixture of phenolphtalein and thymol blue as indicator (Engel and Heins, 1947). Under these conditions, pilot experiments showed a linear relationship between relative dipeptidase concentrations and NaOH consumption up to 9 $\mu$l. (cp. Holter and Løvtrup, 1949).

*Determination of catheptic activity.*

The micromethod of Duspiva (1939) was applied using 1 per cent solutions of nitrocasein or edestin. 10 $\mu$l. of the substrate solutions in acetate buffer at pH 5·3 and 3·8 respectively, were incubated with 5 $\mu$l. of the cell extracts at 40° C. After suitable time, the reaction was stopped with 5 per cent trichloroacetic acid

(20 $\mu$l.), which precipitated the non-digested substrate. The precipitate was then spun down. When using nitrocasein as substrate, an aliquot (20 $\mu$l.) of the supernatant was brought to alkaline pH with NaOH (20 $\mu$l.). The amounts of coloured split products were then determined in a Photovolt microcolorimeter at 420 m$\mu$. This instrument was of the same design as described by Holter and Løvtrup (1949), and Krugelis (1950). In the case of edestin we added Folin-Ciocalteu's reagent to a similar aliquot (20 $\mu$l.), and the amount of split products was determined in a Beckman Model DU, using microcuvettes.

### RESULTS.

The cells in the ascitic fluid represent a mixed population of tumour, mesothelial, white blood cells, and macrophages. In all cases, differential counts indicate that after about two days following inoculation of about $2 \cdot 10^6$ cells, the average percentage of tumour cells amounts to approximately 85 per cent of the total ascites cell numbers (Klein, 1951).

*Protein increase during tumour growth.*

The observed figures for total protein content of tumour homogenates calculated per 10,000 cells are plotted in Fig. 1, A and B. From the 2nd until about the 8th day after inoculation there was a gradual increase in amount of total protein per cell in both Landschütz and Krebs 2 series. After this time the protein content per cell remained at a fairly constant level.

*Proteolytic activity of the ascites fluid and the non-tumorous peritoneal cells.*

The dipeptidase and catheptic activities of the cell-free ascites fluid obtained in this series of ascites tumours were very low. Both activities expressed per microgramme of protein only amounted to about 5 per cent of those of the tumour cells, and showed no significant variations during tumour growth.

The dipeptidase activity of the non-tumorous peritoneal cells obtained by washing the normal peritoneal cavity with saline amounted only to about 0·2 $\mu$l. NaOH per 10,000 cells per hour, using AlaGly as substrate. Since the ratio of tumour cells and non-tumorous cells was constant from the third day after inoculation, it was assumed that variations in enzyme activity of the entire population were due to variations in the enzyme activity of the tumour cells *per se*. This is true under the provision that the non-tumorous exudate cells do not vary their composition and types during the course of tumour growth. The catheptic activity of these exudate cells was also negligible.

Red blood cells are regularly found in many ascites tumours towards the end of their growth period but this cannot explain the observed increase in dipeptidase activities to be reported below. The dipeptidase activity of washed and lysed normal red cells from the commercial mice was found to amount to about 1 $\mu$l. 0·1 M NaOH per $10^6$ cells per hour (AlaGly ; pH 7·7). On the other hand the catheptic activity was very low, amounting to about 2 per cent substrate degradation per $10^6$ cells per hour (edestin ; pH 3·8). Since all tumour samples with macroscopic admixture of blood were discarded, a smallish admixture of blood in cases accepted for investigation can only explain a small part of the observed dipeptidase activities, and will not interfere with the main results.

*Proteolytic activity of washed ascites.*

*Tumour Cells.*—In all series of investigated ascites tumours a gradual increase in the *average dipeptidase activity* per tumour cells was found parallel with the time after inoculation (Fig. 2, upper curves). Similar increase in dipeptidase activity was noted when the observed figures were plotted against the total tumour cell numbers after inoculation (data not reported in detail). Counted on a protein basis, however, the dipeptidase activity was fairly constant throughout the growth of the tumours (Fig. 2, lower curves). In one series of ascites tumours the dipeptidase activity of the tumour cells was tested at a number of stages of tumour growth against several substrates (Table I). With all substrates tested, except GlyTry, a similar increase in the average dipeptidase activity per cell was found with the time passed after tumour inoculation. The most suitable substrate seemed to be AlaGly, which was then used as single substrate in the other series.

TABLE I.—*Dipeptidase Activity of Krebs 2 Tumour Cells at Various Intervals following Inoculation.*

Activities expressed in μl. of 0·1 M NaOH per 70,000 cells in incubation mixture ; time of incubation 2 hours.

| | | Activity in μl. of 0·1 M NaOH. Mean of four determinations. | | | | |
|---|---|---|---|---|---|---|
| Time after tumour inoculation. | Mouse. | dl-Alanylglycine. pH 7·8. | dl-Leucylglycine. pH 7·5. | dl-Leucylglycylglycine. pH 7·5. | Glycyltryptophane. pH 7·5. | dl-Glycylglycine. pH 7·5. |
| 3 days | A | 0·65 | 0·25 | 2·12 | 1·24 | 0·30 |
| | B | 0·68 | 0·71 | 0·95 | 1·35 | — |
| 6 days | C | 3·05 | 1·84 | 3·10 | 1·64 | 1·55 |
| | D | 2·10 | 1·43 | 2·95 | 1·21 | — |
| 13 days | E | 6·55 | 3·06 | 4·85 | 1·04 | 1·60 |

The presence of a dipeptidase inhibitor in the ascitic fluid was observed in two series of pooled Krebs 2 tumour cells. This inhibitor could be removed at least to some extent by repeated washings in 2 per cent NaCl solution. By such means the dipeptidase activity of the cells could be increased by 10 to 40 per cent over the original figures obtained on non-washed cells. Maximum removal was reached after two washings for 3 to 5 minutes each. The occurrence of this inhibitor was not further studied. A similar increase in cathepsin activity was not observed following repeated washing.

TABLE II.—*Range of Catheptic Activities without Added Activator.*

| Ascites tumour. | Substrate. | Percentage substrate degradation per 10,000 cells and 24 hours. |
|---|---|---|
| Ehrlich | Nitrocasein* pH 5·3 | 0·5–1 |
| Landschütz | Edestin pH 3·8 | 3 –10 |
| Krebs 2 | Nitrocasein* pH 5·3 | 1 – 2 |

* Due to insufficient solubility of nitrocasein this substrate could not be used at the catheptic optimum pH 3·8.

At all times after the second day following inoculation, the *catheptic activities* of the three ascites tumours both against nitrocasein and edestin remained at low levels (Table II). Following the addition of cysteine to a final concentration of 2·5 $\times$ $10^{-3}$ M/litre in the incubation mixture, the rate of degradation did increase about 3 times. The average catheptic activities calculated per cell showed no significant changes in the course of tumour growth from the third day after inoculation.

### COMMENTS.

The increase in average protein content per ascites tumour cell during the 2nd to 8th days after inoculation indicates that protein synthesis predominates during this time in comparison with the later stages of ascites tumour growth. The greater rate of increase in cellular protein content during the first few days of the growth coincides with the period of maximum mitotic frequency (Klein and Révész, 1953 ; Lasnitzki, 1953). After the 8th day (Fig. 1) protein synthesis and degradative processes seem to balance each other more or less.

FIG. 1.—Total amount of protein per 10,000 ascites tumour cells during tumour growth. (A) the " Landschütz " variety of Ehrlich ; (B) the Krebs 2 ascites tumour.

The enzymic data suggest a gradual increase in the average dipeptidase activity per tumour cell with the time after inoculation until a certain level has been reached about the 6th to 8th day after inoculation (Fig. 2). The average dipeptidase activity seems, however, roughly proportional to the protein content of the tumour cells. Similar results have been obtained from studies on other material (cp. review by Linderström-Lang, 1952), indicating high dipeptidase values in various growing cells. On the other hand, the average cathepsin activity per ascites tumour cell (measured without added activator(s)), seems very low and constant during ascites tumour growth.

The observed increase in dipeptidase activity may signify a real increase in enzyme content per tumour cell. It might also be effected by a decrease in the amount of enzyme inhibitor(s) possibly present in the cell material. The presence of such dipeptidase inhibitor has only been demonstrated in the *ascitic fluid*, and could at least in part be removed by washing as mentioned above. Similarly, in the case of carboxypeptidases, Ballin and Feinstein (1953) described an inhibitor in the ascitic fluid, probably derived from the white blood cells. Our study, however, provides no information regarding available *intracellular* activators and inhibitors.

The ascites tumours are considered as cell populations including a spectrum of tumour cell generations in different stages of growth. In view of this particular heterogeneity, present techniques permit only conclusions as to the enzymic characteristics of the *average* cell population. More specific information on the enzyme content of ascites tumour cells has been obtained by studies on the topical enzymic patterns of solid forms of ascites tumours (cp. Sylvén and Malmgren, 1955).

FIG. 2.—Dipeptidase activities against AlaGly during the growth of the Ehrlich, Landschütz and Krebs 2 ascites tumours. Means of four determinations. A and C : Activity per 28,000 cells and 3 hours (upper curves), and calculated per microgramme protein and 3 hours (lower curves). B : Activity per 70,000 cells and 30 minutes (upper curve), and calculated per microgramme protein and 3 hours (lower curve).

### SUMMARY.

Serial determinations are reported on the *in vitro* available aerobic catheptic and dipeptidase activities of washed and homogenized Ehrlich, Landschütz and Krebs 2 mouse ascites tumour cell populations at various stages of growth after inoculation. Under present conditions, the total protein content per tumour cell appears to increase until about the 6th to 8th day after inoculation. The average dipeptidase activity measured against *dl*-alanylglycine and calculated per tumour cell shows a similar increase parallel with the time after inoculation and the total tumour cell number. This activity appears to be proportional to the protein content of the tumour cells. During the same time, the average catheptic activity per tumour cell remained low and fairly constant. The proteolytic activities of the ascites tumour fluid, red blood cells and peritoneal cells have been assayed. The presence of a dipeptidase inhibitor in the ascitic fluid is demonstrated.

We are indebted to Dr. George Klein for advice and criticism. The costs have been defrayed by grants to the Cancer Research Division from the Jubilee Fund of His late Majesty King Gustav V and the Swedish Cancer Society.

### REFERENCES.

BALLIN, J. C. AND FEINSTEIN, R. N.—(1953) *Fed. Proc.*, **12**, 173.
DUSPIVA, F.—(1939) *Protoplasma*, **32**, 211.
ENGEL, C. AND HEINS, J.—(1947) *Biochim. biophys. Acta.*, **1**, 190.
HOLTER, H. AND LØVTRUP, S.—(1949) *C. R. Lab. Carlsberg, (Chim.)*, **27**, 27.
KLEIN, G.—(1951) 'The Production of Ascites Tumours in Mice and Their Use in Studies on Some Biological and Chemical Characteristics of Neoplastic Cells.' Uppsala (Almqvist & Wiksell Boktryckeri AB.).
*Idem* AND RÉVÉSZ, L.—(1953) *J. nat. Cancer Inst.*, **14**, 229.
KRUGELIS, E. J.—(1950) *C.R. Lab. Carlsberg, (Chim.)*, **27**, 273.
LASNITZKI, I.—(1953) *Brit. J. Cancer*, **7**, 238.
LINDERSTRÖM-LANG, K. U.—(1952) 'Proteins and Enzymes.' Lane Medical Lectures, **6**, Stanford, California (Stanford University Press).
*Idem* AND HOLTER, H.—(1932) *C.R. Lab. Carlsberg, (Chim.)*, **19**, 1.
NAYYAR, S. N. AND GLICK, D.—(1954) *J. Histochem. Cytochem.*, **2**, 282.
RÉVÉSZ, L. AND KLEIN, G.—(1954) *J. nat. Cancer Inst.* **15**, 253.
SYLVÉN, B. AND MALMGREN, H.—(1955) *Exp. Cell Res.* **8**, 575.